# Evaluation of a cIEF Fractionation Workflow for Offline MS Analysis of Charge Variants of the Monoclonal Antibody Matuzumab

**DOI:** 10.1002/elps.8108

**Published:** 2025-02-18

**Authors:** Antonia Wittmann, Yannick Wilke, Nicolas Grammel, Hermann Wätzig

**Affiliations:** ^1^ Institute of Pharmaceutical Technology and Biopharmaceutics TU Braunschweig Braunschweig Germany; ^2^ Institute of Medicinal and Pharmaceutical Chemistry TU Braunschweig Braunschweig Germany; ^3^ Alvotech Hannover GmbH Hannover Germany

**Keywords:** electrophoresis, fractionation, imaged capillary isoelectric focusing, mass spectrometry, Matuzumab, monoclonal antibody

## Abstract

Biological drugs like monoclonal antibodies require careful analysis and characterization to ensure product quality, safety, and efficacy. Charge variants of the molecule are of key interest and are analyzed using imaged capillary isoelectric focusing (icIEF). However, deeper characterization of these variants poses challenges. Two workflows for their characterization exist: an ion‐exchange chromatography method for variant collection before mass spectrometry (MS) analysis, which is labor‐intensive, and direct coupling of CE to MS, which allows detailed structural characterization but has limitations, for example, due to incompatibilities with ES ionization using high BGE concentrations. This study evaluates a platform that fractionates charge variants for offline MS analysis. The suitability of a procedure in which analytical icIEF methods are converted into preparative cIEF fractionation methods by increasing the sample concentration and adding 20 mM arginine as a cathodic spacer was tested. After chemical mobilization and fraction collection, the identity of the fractions was determined by fluorescence measurement and reinjection of the protein‐containing fractions, using the previously developed analytical icIEF method. MS was subsequently performed. The general suitability of the workflow was demonstrated using Matuzumab. Transferring the analytical method from a concentration of 0.2 to 1.2 mg/mL in fractionation yielded an identical number of peaks and visually comparable peak profiles. The preparative separation took 50 min, with an additional 25 min for mobilization and 45 s per fraction collection, totaling approximately 2.5 h. Verification of charge variant isolation was straightforward via analytical icIEF. Following fractionation, MS allowed for the identification of the main peaks. Preliminary results with other antibodies indicated that the concentration range for MS experiments needs further investigation. Future work will aim to optimize sensitivity, selectivity, analysis time, and reproducibility.

## 1 Introduction

Biologics have become the most important and innovative group of pharmaceuticals. Among these drugs, monoclonal antibodies are currently the most important, but others such as RNA and DNA, viral vectors, and cell preparations are also gaining in importance.

Biologics are complex. Typically, they do not consist of one single species but rather a variety of very similar ones with possibly similar effects, which form the pharmaceutical‐related compounds. In addition, by‐products from production, for example, host‐cell proteins, degradation products, and glycosylation variants with various effects and side effects, need to be reliably characterized [1, 2, 3, 4, 5, 6, 7, 8, 9, 10, 11, 12, 13]. Typically, several dozen compounds need to be separated and characterized. Therefore, there is a great demand for reliable platform methods.

Imaged capillary isoelectric focusing (icIEF), capillary zone electrophoresis (CZE) and ion exchange chromatography (IEX) are highly effective and widely used techniques for analyzing such variants of monoclonal antibodies [6, 7, 14].

Capillary electrophoresis coupled to mass spectrometry (CE–MS) is one of the preferred techniques to characterize charge variants as it combines high separation efficiency in the first dimension with mass information in the second dimension [15, 16, 17, 18]. However, CE–MS is still more useful in laboratories with an experienced team. For broader application, other approaches are needed that can be established quickly [15].

Direct combination of icIEF and MS is challenging because common icIEF reagents such as urea or methylcellulose can lead to ion suppression in ESI in direct CE–MS coupling [4]. Therefore, MS‐compatible icIEF separation conditions have been developed. Capillary coatings that do not require the addition of methyl cellulose (MC) can be utilized. In addition, the urea used for solubilization in iCIEF can be replaced with formamide [4, 17].

In recent years, a new approach was introduced that allows for easy collection of charge variants for offline characterization. In the workflow, charge variants are focused by icIEF and then mobilized chemically into wells of a 96‐well plate. This workflow allows for offline characterization of charge variants by several techniques including CE, MS, and even for binding assays (SPR). It allows flexibility in the use of different reagents and excipient, as these are reliably removed during the preparative separation. We were interested to characterize the capability of the platform to provide sufficient information for complex biologics in a rapid and reliable fashion. For this work, we leveraged a well‐characterized antibody, Matuzumab.

## 2 Materials and Methods

### 2.1 Instrumentation

All icIEF analyses (analytical and preparative) were performed using a MauriceFlex instrument (ProteinSimple, a Bio‐Techne brand, San Jose, CA, USA). The entire working procedure was based on information from the manufacturer: User Guide for Maurice, Maurice C., Maurice S. and MauriceFlex, Revision D, PN 046–295, 2023. Default conditions for temperature settings were used, and the temperature was kept at around 10°C for the samples. The capillary was automatically calibrated with the fluorescence calibration standard before sample measurements. Data were analyzed using Compass for iCE 4.0.0 and 4.0.1, which was also developed by ProteinSimple, a Bio‐Techne brand.

All fluorescence measurements of the well plates were carried out using an Infinite 200 PRO instrument (Tecan Group Ltd. Männedorf, Switzerland) using top‐read, excitation wavelength 280 nm and emission wavelength 350 nm, and Tecan i‐control, version 2.0.10.0 as software.

### 2.2 Reagents

Ultrapure water (electrical conductivity: 0.055 µS/cm) was freshly produced by an Arium pro VF system (Sartorius AG, Göttingen, Germany). Pharmalytes (PLs) (Cytiva Global Life Sciences Solutions USA LLC, Marlborough, MA 01752, USA), lyophilized pI‐markers (reconstituted with 210 µL water to 100 µg/mL and aliquoted), MC 1% and 0.5%, l‐arginine (500 mmol/L), anolyte (80 mmol/L phosphoric acid in 0.1% MC), catholyte (100 mmol/L NaOH in 0.1% MC), Maurice cIEF fluorescence calibration standard, lyophilized urea (reconstituted with 320 µL water to 10 mol/L), ammonium acetate (2 M, for mobilizer solution), and cIEF System Suitability Kit, for analytical icIEF experiments (Maurice cIEF System Suitability Peptide Panel and System Suitability Test Mix), were provided by ProteinSimple, a Bio‐Techne brand (San Jose, CA, USA).

Crystalline urea (>98%) was provided by Sigma‐Aldrich Chemie GmbH (Taufkirchen, Germany). PL 3–10, 5–8, and 8–10.5 were used for verification experiments and obtained from GE Healthcare, Bio‐Sciences AB, Sweden. Matuzumab was received as a gift from Merck KGaA (Darmstadt, Germany).

### 2.3 Sample Preparation Analytical and Preparative Separation

#### 2.3.1 Analytical icIEF

For analytical icIEF, the Maurice cIEF cartridge was used. This cartridge had a capillary length of 50 mm, with dimensions of 100 µm inner diameter and 200 µm outer diameter and was made of fused silica coated with fluorocarbon. Sample injection was performed over 55.0 s using vacuum. Detection was carried out using native fluorescence with excitation at 280 nm and emission between 320 and 450 nm and an exposure time of 10 s. The voltage program for analytical separation of Matuzumab involved applying 1500 V for 1.0 min, followed by 3000 V for 11.0 min.

The analytical icIEF method was based on a literature method and slightly modified [14] (Table 1).

**TABLE 1:** Preparation of capillary isoelectric focusing (cIEF) solution with sample and reagents: Analytical separation (based on [14]).

Reagents	V [µL]	Final concentration
Urea^a^	—	2 mol/L
DI water	55.3	
1% MC	35	0.35% (V/V)
Pharmalyte 8–10.5	2	2% (V/V)
Pharmalyte 5–8	2	2% (V/V)
pI marker 6.14	2	2% (V/V)
pI marker 9.5	2	2% (V/V)
Matuzumab stock (11.75 mg/mL)	1.7	0.2 mg/mL
Final volume	100	

^a^ 12.02 mg urea was weighed into a microcentrifugation tube.

Abbreviation: MC, methyl cellulose.

The MauriceFlex cIEF fractionation cartridge, which has a capillary length of 50 mm and an inner diameter of 320 µm, is made of fused silica coated with fluorocarbon. The injection process lasts for 20.0 s and is performed using a vacuum. For detection during separation, native fluorescence is measured with an excitation wavelength of 280 nm and an emission wavelength of 320–450 nm. The exposure time is 0.2 s, and the detection interval is set to 5.0 min. During mobilization and fraction collection, the native fluorescence is similarly measured, with an exposure time of 0.2 s and a detection interval of 1.0 min.

In preparative separation of Matuzumab, the voltage program consists of applying 500 V for 10.0 min, followed by 1000 V for another 10.0 min, and then 2000 V for 30.0 min.

In all experiments, the reagents, which include 0.5% MC, anolyte, catholyte, fluorescence calibration standard, ultrapure water, and 5 mM ammonium acetate for fractionation only, as well as the cartridges for analytical or preparative icIEF, were used according to the manufacturer's instructions (Table 2).

**TABLE 2:** Preparation of capillary isoelectric focusing (cIEF) solution with sample and reagents: Preparative separation.

Reagents	V [µL]	Final concentration
Urea 10 M	30	2 mol/L
DI water	34.2	
1% MC	52.5	0.35% (V/V)
Pharmalyte 5–8	3	1% (V/V)
Pharmalyte 8–10.5	3	1% (V/V)
pI marker 9.50	3	1% (V/V)
pI marker 6.14	3	1% (V/V)
l‐Arginine (500 mM)	6	20 mmol/L
Matuzumab stock (11.75 mg/mL)	15.3	1.2 mg/mL
Final volume	150	

Abbreviations: MC, methyl cellulose; pI, isoelectric point.

#### 2.3.2 Sample Preparation Analytical and Preparative Separation

Urea was prepared either from a concentrated stock solution, which was made by dissolving lyophilized urea into a 10 M solution, or by directly weighing the required amount of crystalline urea into a microcentrifuge tube, then dissolving it in the total amount of water before adding the other reagents. Aliquots of antibodies and pI‐markers were stored at −23°C and were thawed at room temperature. After all reagents and the respective antibody were pipetted into the samples, the samples were vortexed and then centrifuged for 3.0 min at 12 300 × *g*.

Samples were not used for more than 24 h due to the known instability caused by urea. The desired amount of the sample was then transferred to a 96‐well plate. To remove air bubbles from the sample, the 96‐well plate was centrifuged for 1.0–2.0 min at 1000 × *g*, and the sample surfaces were treated with isopropyl alcohol to eliminate bubbles. System suitability tests for analytical icIEF experiments were conducted according to the manufacturer's recommendations.

For fraction collection after preparative separation, 30 µL of ammonium acetate (prepared by dissolving 20 µL of 2 M ammonium acetate in 8.0 mL of ultrapure water) was used as a mobilizer solution and pipetted into the wells, starting with row B. Row A contained only the samples to be fractionated, without ammonium acetate. After fractionation, the plates were stored at −23°C. The stability of the fractions after one or more freeze‐thaw cycles was not evaluated.

Fluorescence measurements were performed to determine the position of the protein‐containing fractions on the 96‐well plate for subsequent mass spectrometry (MS). If any air bubbles were visible within the wells, they were removed by centrifugation for 1.0–2.0 min at 1000*g* and by vaporizing isopropyl alcohol (Table 3).

**TABLE 3:** Preparation of capillary isoelectric focusing (cIEF) solution with sample and reagents: Analytical separation of the reinjected collected fractions.

Reagents	V (µL)^b^	Final concentration	V (µL)^c^
Urea^a^	—	2 mol/L	
DI water	14.8		639.36
1% MC	14	0.35% (V/V)	604.8
Pharmalyte 5–8	0.8	2% (V/V)	34.56
Pharmalyte 8–10.5	0.8	2% (V/V)	34.56
pI marker 9.50	0.8	2% (V/V)	34.56
pI marker 6.14	0.8	2% (V/V)	34.56
Finale volume master mix	32		1382.4
Addition of reference sample or fraction^d^	8		
Final volume	40		

^a^ 207.31 mg urea was directly weighed into an Eppendorf tube and dissolved in the entire volume of water.

^b^ Volume of reagents for the desired amount for one sample of 40 µL.

^c^ Volume calculation of the total quantity of a master mix. As three series of 12 fractions each were analyzed, the 36‐fold amount from the second row was pipette, plus an excess of 20% to compensate for eventual losses. For the single analyses, 32 µL Master Mix and 8 µL analyte solution were merged.

^d^ 8 µL of a protein‐containing well after fractionation or 8 µL reference sample with a final concentration of 0.05 mg/mL Matuzumab (non‐fractionated).

#### 2.3.3 Verification Experiments Matuzumab

##### 2.3.3.1 Sample Preparation Verification Experiments

Before verification experiments, system suitability tests were performed. The same Matuzumab sample was separated (preparative mode) three times and collected each time in a new plate with fresh mobilization solution. A post‐run cartridge cleanup was performed after each preparative separation. Several fractions from the three fractionations were each verified in triplicate using analytical icIEF. Either 0.05 mg/mL of Matuzumab was used as a reference, or Matuzumab fractions obtained from preparative icIEF were used without calculating their concentration. These fractions were subjected to a 1:5 dilution prior to verification.

The verification experiments were performed based on the previously used analytical icIEF separation conditions. However, the methods were slightly modified as described below.

First, the Master Mix was prepared by pipetting all reagents as shown in the table above and was then vortexed and centrifuged (3.0 min 12 300 × *g*). The 96‐well plates (stored at −23°C) with the fractions were thawed at room temperature. A volume of 8 µL of each of the protein‐containing fractions were added to 32 µL of Master Mix in a new 96‐well plate and mixed in the well by pipetting up and down.

In addition, a reference sample was prepared with the non‐fractionated antibody, consisting of 32 µL Master Mix and 8 µL of a dilution of the antibody stock solution. The final concentration of Matuzumab was 0.05 mg/mL, calculated on a total volume of 40 µL sample. The concentration of the reference was reduced compared to the previous analytical icIEF in order to adapt it to the concentration of the fractions. Fluorescence exposure time for detection was increased (20, 50, 80, and 100 s were measured for each fraction, 10 s was default in the previously described experiments) due to reduced sample concentration in each fraction after fractionation.

### 2.4 Mass Spectrometric Investigations of Collected Fractions

Reversed‐phase separation of 10 µL of the collected fractions was carried out using an Ultimate 3000 RSLC system (Thermo Scientific, Waltham, MA, USA) with a Waters MassPREP Cartridge (1000 Å, 20 µm, 2.1 mm × 10 mm). The column was initially equilibrated with 84% mobile phase A (0.1% Formic acid in water) and 16% mobile phase B (0.1% formic acid in acetonitrile) for 3 min. Elution was achieved with a linear gradient of 16%–90% B over 2 min at 55°C at a flow rate of 0.2 mL/min. Data were acquired using a UV detector at the wavelength of 214 nm. Mass spectrometric analysis was performed in a positive ion mode on an Ultra‐High‐Resolution maXis QTOF mass spectrometer equipped with an ESI source. For data acquisition, end plate offset was set at 500 V, capillary voltage was set at 3500 V, the nebulizer was set at 3 bar, dry gas was set at 9.0 L/min, and dry temperature was set at 190°C. Mass spectra were acquired from 1200 to 5200 *m/z* with a spectra rate of 0.25 Hz. ESI‐L Low Concentration Tuning Mix (Agilent Technologies) was used for the internal calibration. ESI mass spectra were deconvoluted with the DataAnalysis (Bruker) software using the maximum entropy algorithm.

## 3 Results and Discussion

### 3.1 General Approach

The monoclonal antibody Matuzumab was chosen as test analyte throughout this project. Matuzumab is a humanized monoclonal antibody against EGFR and developed for cancer therapy, but its clinical development was discontinued [19, 20, 21].

First, analytical icIEF separation methods were developed and then transferred to preparative icIEF methods. Afterward, a verification step was introduced for the collected fractions. At first, the fluorescence within the 96‐wells plates was measured to determine the protein containing fractions. In a second step, these fractions were analyzed for peak identity and purity using the same previously developed analytical icIEF methods. Subsequently fractions were analyzed by MS, although other assays could be performed as well.

### 3.2 Analytical and Preparative Separation

**FIGURE 1:**
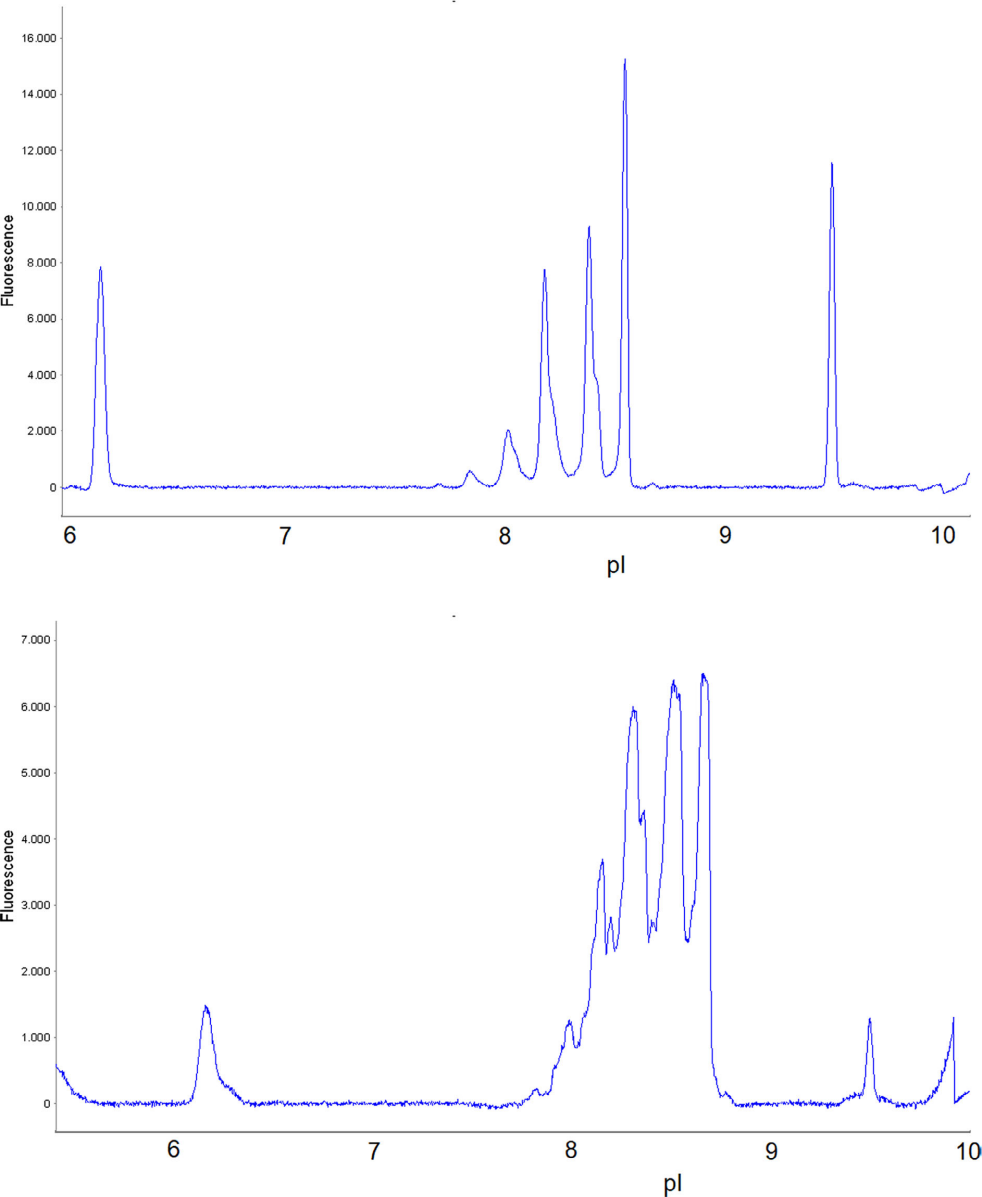
Electropherogramms of Matuzumab: analytical iciEF (top), preparative icIEF (bottom).

The analytical separation of Matuzumab and related substances was accomplished in a straightforward manner (Figure 1, top). As the separation conditions are close to the standard conditions suggested by the manufacturer, the method development took less than 1 day. To test the method, one sample was injected three times. This was repeated with a newly prepared sample three times. Identical separation profiles were obtained by overlay electropherograms of successive runs.

The development of the preparative method was straightforward as well using the MauriceFlex cIEF Fractionation Cartridge. The number of peaks was the same as in the analytical separation, and the peak profile was very similar (Figure 1, bottom).

In general, higher sample concentrations and a larger inner capillary diameter of 300 µm are used for the preparative separation, and l‐arginine is added as a cathodic spacer. The chemical mobilization is intended to prevent urea, the ampholytes and methylcellulose from entering the fractions to any significant extent so that mass spectrometric measurements of the fractions are possible after a small sample pretreatment.

Here, too, the standard conditions from the Maurice cIEF Method Development Guide only had to be adapted accordingly. In this case, the sample concentration, the injection time, and the voltage program were adjusted (see Experimental). The voltage was increased to accelerate the separation. However, to avoid excessive reduction in separation efficiency or damage to the capillary coating due to Joule heating, the current should not exceed 90 µA. The focusing time was adjusted until the peak profile remained visually constant.

The experiments were highly reproducible. Differences in pI were less than 0.07 for all major peaks throughout the whole series of experiments between the analytical and preparative experiments. Comparing the pI for analytical and preparative, there was an apparent shift of 0.1, easily explainable by the change in peak shape (Figure 1). This indicates that the separation mechanism is largely maintained from analytical to preparative separation. However, the separation efficiency is lower in the preparative separation, which is probably partly due to the larger capillary diameter and electrodispersive forces due to the very high analyte concentrations in the focused zones.

The fractions were collected in 96‐well plates, each well containing 5 mM ammonium acetate solution, into which the fractions were collected. The preparative separation took 50 min, and the entire process including fractionation required approximately 2.5 h. The plates can be easily frozen and stored for further experiments. This is usually possible with monoclonal antibodies, but the stability of other bioanalytes must be considered on a case‐by‐case basis.

All plates were checked within 10 min using a fluorescence reader (see Experimental) to determine which of the fractions contained a significant amount of protein above the blank value. Only those fractions were considered for further experiments.

**FIGURE 2:**
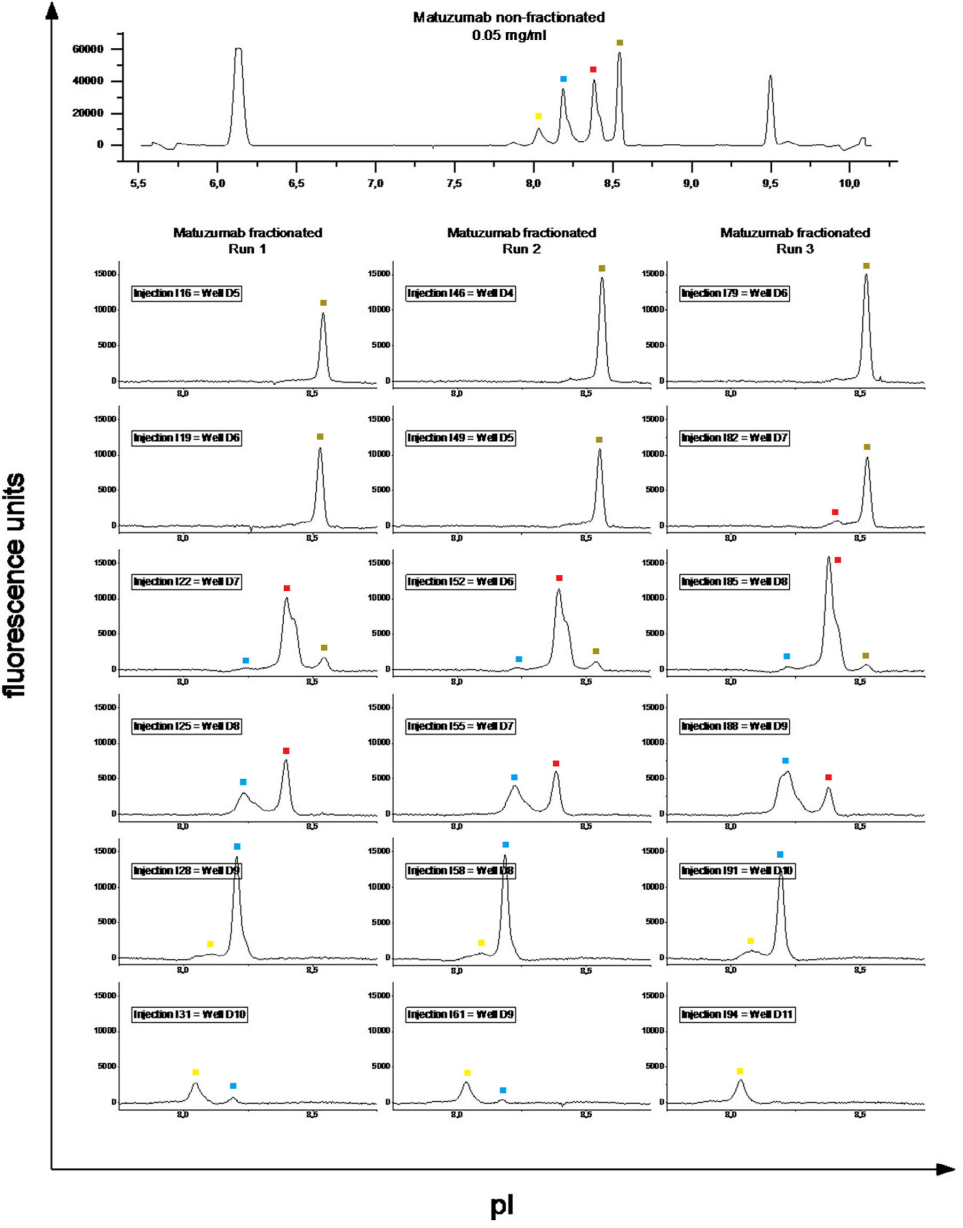
The same Matuzumab sample was separated (preparative mode) three times and collected each time in a new plate with fresh mobilizer solution. Several fractions of the three fractionations were each verified in triplicate using analytical icIEF (only one of three runs is shown). Corresponding species in the fractions are marked by the same colored dot.

A more comprehensive verification experiment was also performed. Matuzumab was separated (preparative mode) three times and collected each time in a new plate with fresh mobilizer solution. Several fractions from each of the three fractionation runs were verified in triplicate using analytical icIEF (Figure 2). All charge variants of Matuzumab were recovered in the fractions with comparable peak profiles and positions per fraction(see also Supporting Information section S1).

Across the three fractionation runs, a small shift (1–2 wells) in fraction deposition location was observed. However, by verifying the fractions by analytical icIEF, the matching fractions can be assigned easily. A verification experiment consists of several single analytical icIEF runs. In Figure 2, 18 runs are shown, but three times as much experiments were performed. Therefore, this type of experiment takes about 1 or 2 days, depending of the number of fractions considered and the number of repetitive experiments. This is not intended for routine analysis, but for one‐time confirmation and validation of the rapid results of the fluorescence reader.

### 3.3 MS and Sample Concentration

**FIGURE 3:**
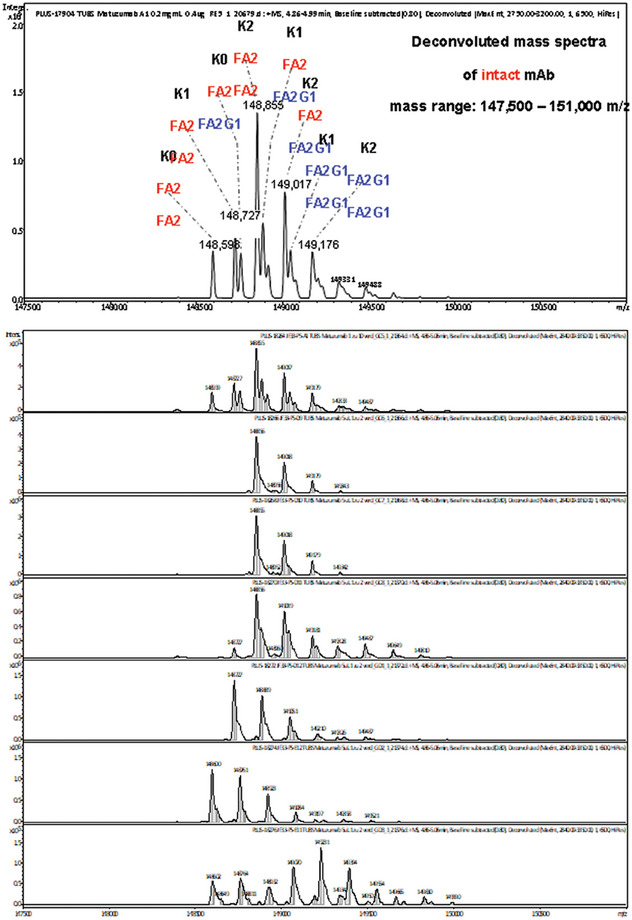
Mass spectra of Matuzumab. Non‐fractionated (top), Matuzumab fractions after preparative icIEF (below).

The mass spectra of the protein‐containing fractions were successfully obtained and compared to the spectrum of Matuzumab without prior separation. The various fractions contained variants differing in the terminal lysine where K2, K1, and K0 variants could be easily distinguished in the fractions. These C‐terminal lysine variations are commonly observed in biopharmaceutical monoclonal antibodies, and they can be important as they are often sensitive to the production process [22]. The expected glycosylation pattern FA2 and A2G1 (according to Oxford nomenclature) were also found (Figure 3). These patterns and their batch‐to‐batch conformity are critical quality attributes (CQAs) [23], and antibody glycoyslation has been extensively discussed in the scientific literature (see recommended recent references: [24, 25]). In addition, however, a fraction with one or more additional acidic species was obtained that have not been reported previously. This shows the additional value of this two‐dimensional concept.

### 3.4 Comparison of the Performance of Various Instrumental Set‐Ups

The approach presented is very robust and reliable. It provides a lot of additional structural information about the nature of the biological molecules that make up a biological drug. Other recent approaches such as the analysis of adeno‐associated viruses (AAVs), a therapeutic Fab fragment, and bispecific antibodies by CE–MS and the introduction of new interfacing options underline this excellent performance [26, 27, 28, 29, 30].

Other approaches offer complementary information with comparable resolving power and structural information. Using an alternate technical approach, the NIST antibody was successfully characterized at a concentration of 1 mg/mL [31]. A hydrophobic interaction chromatographic approach combined with zone electrophoresis (HIC‐CZE‐UV) managed to separate almost 30 substances. Although it is hardly possible to obtain structural information with a UV detector, the information on the number of fractions can be combined very well with other findings [1].

## 4 Conclusion and Outlook

The general suitability of preparative icIEF with MS offline coupling was demonstrated using Matuzumab. Similar to previous experiences with iciEF, the development of the analytical icIEF method for Matuzumab (0.2 mg/mL) was easily accomplished. The transfer to the cIEF fractionation mode with higher concentrations (approximately 2.0 mg/mL) and modified capillary dimensions with arginine as cathodic spacer was easy to implement as well. Using default conditions, fractionation methods can be developed in 1 or 2 days. Each fractionation run takes approximately 2.5 h. The fluorescence measurement of the plate after fractionation provides a good overview of protein containing wells within 10 min. Analytical icIEF is a suitable tool for the verification of individual fractions and takes approximately 1 or 2 days. However, a new iciEF method was recently published that can dramatically decrease the focusing time and should be explored for fraction verification [32]. Yet verification is only needed once during method development or changes in method parameters. The quality of a fractionation method can be easily assessed using the previously established analytical icIEF method.

Analysis time, reproducibility, selectivity, and sensitivity will be optimized in the future to obtain well‐suited platform methods. We will likely focus on the latter two parameters. To increase sensitivity, there are three major ways to increase the injected sample amount for icIEF. First, the sample volume can be increased. However, this also means reducing the volume of the master mix. This approach may require adjustments to the master mix solutions, which will probably need to be more concentrated. Second, one can start with a higher sample concentration. The manufacturer recommends 0.5–2 mg/mL in the final injected solution, that is, in the solution that also contains the master mix (see Experimental). Although 0.5 mg/mL is sufficient in favorable cases, 2 mg/mL should be used as a guideline. Moreover, this means that the actual sample concentration before mixing with the master mix should be in the range of 5–10 mg/mL (or higher, if possible). These figures refer only to the subsequent mass analysis. Less (or more) material may be required for other uses of the collected fractions [27]. One can also consider options to increase protein concentration. For example, Vivaspin columns can preferably be used to concentrate proteins, if they are not too sensitive to mechanical stress. Depending on the protein or biologic in general, the protocol must be carefully selected to avoid protein degradation while maximizing protein concentration. Finally, sample preparation prior to MS can also be optimized. As the entire approach is new, many new ideas can certainly emerge here.

The separation conditions must be adapted to the sufficient sample concentration. Even if the peak profile of the preparative icIEF does not look as good as that of the analytical one, this does not usually matter. All that is required is sufficient separation so that the subsequent MS step is as meaningful as possible.

Options to improve selectivity include narrower ampholyte ranges and various solubilizer reagents including surfactants such as polysorbate and sulfabetaine [33, 34]. It will be interesting to see if these options work differently depending on the pH range used.

Then the fractionation can be optimized, for example, using a higher number of fractions. Additional measures prior to analysis such as sample pretreatment including the use of enzymes such as Pfu, carboxypeptidase, sialinase, and glycosidases can be considered. Investigating stressed samples under conditions like oxidation, light exposure, temperature variation, and shaking is also valuable.

This cIEF fractionation with offline MS approach provides excellent selectivity and sensitivity for thoroughly investigating all charge variants of pharmaceutical monoclonal antibodies. The straightforward method development and reliable technical setup enable platform methods to effectively characterize these crucial substances during drug development (Supporting Information).

## Conflicts of Interest

The authors declare no conflicts of interest.

## Supporting information



Supporting Information

## Data Availability

The data that support the findings of this study are available from the corresponding author upon reasonable request.
