# Supplementary material for: Evaluation of a cIEF Fractionation Workflow for Offline MS Analysis of Charge Variants of the Monoclonal Antibody Matuzumab
Source: Electrophoresis. 2025 Feb 18;46(3-4):240–9. doi: 10.1002/elps.8108 (PMC11865688; doi:10.1002/elps.8108)
Supplement: Supplementary file 1 — Supporting Information [file ELPS-46--s001.docx]

**Supporting Material**

**Concentration determination Matuzumab**

**Instrument**

- **UV-Vis spectrophotometer SPECORD 40; Analytik Jena GmbH + Co. KG (Jena, Germany)**
  - determining Matuzumab stock concentration via Pierce BCA Protein Assay Kit; Thermo Fisher Scientific Inc. (Waltham, MA, USA)
    - the standard protocol from the kit (working range 20-2000 µg/mL) was used
  - Software: WinASPECT, version 2.5.0.0; Analytik Jena GmbH + Co. KG (Jena, Germany)

**Reagents**

- Pierce BCA Protein Assay Kit (Lot: UC281368); Thermo Fisher Scientific Inc. (Waltham, MA, USA)
- Matuzumab was received as a gift from Merck KGaA (Darmstadt, Germany)

Concentration determination (: Via Pierce BCA Protein Assay Kit with BSA as reference standard. The standard protocol from the kit (working range 20-2000 µg/mL) was used and a correction factor of x*(1/1.09) (x = uncorrected antibody concentration), derived from an absorption ratio table, found in the assay instructions for human IgGs vs. BSA, was applied to the results. A polynomial fit of the 3rd degree was used for the calibration curves (compare Thesis Holger Zagst, Braunschweig 2024).
